# Characterization of SARS-CoV-2 different variants and related morbidity and mortality: a systematic review

**DOI:** 10.1186/s40001-021-00524-8

**Published:** 2021-06-08

**Authors:** SeyedAhmad SeyedAlinaghi, Pegah Mirzapour, Omid Dadras, Zahra Pashaei, Amirali Karimi, Mehrzad MohsseniPour, Mahdi Soleymanzadeh, Alireza Barzegary, Amir Masoud Afsahi, Farzin Vahedi, Ahmadreza Shamsabadi, Farzane Behnezhad, Solmaz Saeidi, Esmaeil Mehraeen, Shayesteh Jahanfar

**Affiliations:** 1 Iranian Research Center for HIV/AIDS, Iranian Institute for Reduction of High Risk Behaviors, Tehran University of Medical Sciences, Tehran, Iran; 2 Department of Global Health and Socioepidemiology, Graduate School of Medicine, Kyoto University, Kyoto, Japan; 3 Chronic Respiratory Disease Research Center, Masih Daneshvari Hospital, Tehran, Iran; 4 School of Medicine, Tehran University of Medical Sciences, Tehran, Iran; 5 Ophthalmology Resident at Farabi Hospital, School of Medicine, Tehran University of Medical Sciences, Tehran, Iran; 6 School of Medicine, Islamic Azad University, Tehran, Iran; 7 Department of Radiology, School of Medicine, University of California, San Diego, CA USA; 8 Department of Health Information Technology, Esfarayen Faculty of Medical Sciences, Esfarayen, Iran; 9 Department of Virology, School of Public Health, Tehran University of Medical Sciences, Tehran, Iran; 10 Department of Nursing, Khalkhal University of Medical Sciences, Khalkhal, Iran; 11 Department of Health Information Technology, Khalkhal University of Medical Sciences, 1419733141 Khalkhal, Iran; 12 Department of Public Health and Community Medicine, Tufts University School of Medicine, Boston, MA USA

**Keywords:** Variants, Strains, Genomic diversity, Characterizations, SARS-CoV-2, COVID-19

## Abstract

**Introduction:**

Coronavirus Disease-2019 (SARS-CoV-2) started its devastating trajectory into a global pandemic in Wuhan, China, in December 2019. Ever since, several variants of SARS-CoV-2 have been identified. In the present review, we aimed to characterize the different variants of SARS-CoV-2 and explore the related morbidity and mortality.

**Methods:**

A systematic review including the current evidence related to different variants of SARS-CoV-2 and the related morbidity and mortality was conducted through a systematic search utilizing the keywords in the online databases including Scopus, PubMed, Web of Science, and Science Direct; we retrieved all related papers and reports published in English from December 2019 to September 2020.

**Results:**

A review of identified articles has shown three main genomic variants, including type A, type B, and type C. we also identified three clades including S, V, and G. Studies have demonstrated that the C14408T and A23403G alterations in the Nsp12 and S proteins are the most prominent alterations in the world, leading to life-threatening mutations.The spike D614G amino acid change has become the most common variant since December 2019. From missense mutations found from Gujarat SARS-CoV-2 genomes, C28854T, deleterious mutation in the nucleocapsid (N) gene was significantly associated with patients' mortality. The other significant deleterious variant (G25563T) is found in patients located in Orf3a and has a potential role in viral pathogenesis.

**Conclusion:**

Overall, researchers identified several SARS-CoV-2 variants changing clinical manifestations and increasing the transmissibility, morbidity, and mortality of COVID-19. This should be considered in current practice and interventions to combat the pandemic and prevent related morbidity and mortality.

## Introduction

In December 2019, a novel coronavirus (CoV) emerged from Hubei province in China among people visiting the Huanan seafood market in Wuhan. The virus is transmitted through human-to-human contact and rapidly spread across the world, and soon turned into a pandemic [1, 2]. Its symptoms can be divided to two main groups of majors (fever, cough, dyspnea) and minors (anosmia, dysgeusia, headache, gastrointestinal symptoms, skin lesions) [3–6]. Ever since, it has caused worldwide social and economic disruption. There are no universally recognized clinically useful antiviral drugs against COVID-19 so far (actually remdesivir showed good efficacy in many trials, but it needs to be discussed more). Although there are several effective vaccines to prevent infection with SARS-CoV-2, efforts to develop medications and vaccines are continuing [7].

It appeared that there are several variants of SARS-CoV-2. Since the beginning of the pandemic; there have been tremendous efforts to determine the genetic diversity of virus and discover the variations such as immune targets change (such as the spike glycoprotein), primer-binding and probe-binding sites change (which can reduce the sensitivity of diagnostic tests), and genetic variations (which might affect transmissibility and virulence) [8–11].

SARS-CoV-2 genome consists of approximately 29,903 nucleotides and organized in the following order from 5' to 3': open reading frame (ORF) 1ab (replicas), structural spike glycoprotein (S), ORF3a protein, a structural envelope protein (E), structural membrane glycoprotein (M), ORF6 protein, ORF7a protein, ORF7b protein, ORF8 protein, structural nucleocapsid-phosphoprotein (N), and ORF10 protein. ORF1ab is a large polyprotein (∼ 21,291 nucleotides) encoding sixteen non-structural proteins: leader protein, nsp2, nsp3, nsp4, 3C-like proteinase, nsp6, nsp7, nsp8, nsp9, nsp10, RNA-dependent RNA polymerase (RdRp), helicase, 3'–5' exonuclease, endoRNAse, 2'-o-ribose methyltransferase, and nsp11 [12].

Different variants have been discovered besides the wild-type, with a certain amount of nucleotide deletion in the ORF8 (which is the biological function of the ORF8 protein in SARS-CoV-2 remains unclear). The most known one is the ∆382 variant, a 382-nucleotide deletion in the ORF7b and ORF8, removing its transcription-regulatory sequence (this omission stops ORF8 transcription). This variant was successfully early transmitted during the epidemic, but was unknown until March 2020. These different variants have been found around the world in countries such as Bangladesh (345 nucleotides), Australia (138 nucleotides), and Spain (62 nucleotides). An identical Δ382 variant was also detected in February 2020 in a traveler who returned from Wuhan, China, to Taiwan [13, 14].

In the last pandemic of 2002–2003, the SARS-CoV was responsible for zoonotic transmission from civets to humans. After a short time, the virus's wild-type mutated, and a new variant emerged, which had a 29-nucleotide deletion in the ORF8, known as the Δ29. Subsequently, there were some reports of 82-nucleotide deletion and 415-nucleotide deletions in the same region. The influence of these deletions on the pandemic is still unknown. But some in vitro studies have shown that Δ29 replicates less efficiently and causes milder clinical illness than the wild-type [15, 16].

The relations between the magnitude of nucleotide deletion in ORF8 with its virulence and the ORF8 function are still unknown. However, a recent study suggested that ORF8 mediates immune evasion by downregulating MHC-I molecules. In vitro studies have also shown that these deletions do not affect replicative fitness, but it can affect the transcription of some essential and defensive regions such as ORF6 and N genes (known as SARS-CoV interferon antagonists); thus, it can create a more fragile variant compared to the wild-type [17–19].

Besides ORF8, other genome parts can be affected by the mutations, and new variants could emerge. Studies have revealed that the highly mutable spike (S) protein of the virus is associated with the elevated human-to-human transmission rate through interaction with the host's ACE2 receptor. S protein is one of the well-characterized proteins of the Coronaviridae family; this ∼ 1255 amino acid transmembrane protein helps the virus to attach and enter the host [12, 20].

There are also reports about the mutations in other parts such as nsp2 and nsp12 (RdRp). The SARS-CoV-2 nsp12 is RNA-dependent RNA polymerase (RdRp) consisting of 932 amino acids located in the polyprotein, from 4393 to 5324 aa. Structurally, the SARS-CoV-2 nsp12 protein is categorized into N-terminal (1–397aa) and a polymerase domain (398–919aa). These mutations have been observed in patients from India, Germany, and Iran [20].

In this article, we aimed to report on and compare the morbidity and mortality of the different variants of SARS-CoV-2 with that of the wild-type to realize whether they could be an immense threat to humans.

## Methods

This study is a systematic review of current evidence conducted in September 2020. The authors aimed to study the effect of different variants of SARS-CoV-2 on mortality and morbidity. Our study is consistent with the Preferred Reporting Items for Systematic Reviews and Meta-Analyses (PRISMA) checklist to ensure reported results' reliability and validity.

### Sources of data

We retrieved all the relevant papers and reports published in English from December 2019 to September 2020 through a systematic search using keywords in the online databases of PubMed, Web of Science, Scopus, and Science Direct. We updated our search on late February 2021. Our search strategy employed multiple combinations of keywords, as follows:

- "SARS-CoV-2" OR "Coronavirus" OR "COVID-19" OR "2019-nCoV" OR "Novel Coronavirus"[Title/abstract]
- "Variants "OR "Variation" OR "Strains" OR "Types" OR "Minority Variants" OR "Genomic Variants" OR "Genetic Variation" OR "Genomic Diversity"OR "Characterizations" [Title/abstract]
- [A] and [B]

### Selection of the study

Three independent investigators reviewed the extracted papers' full text and selected the most pertinent papers according to the eligibility criteria. Then we pulled the relevant data and organized them in some tables. We included the original and peer-reviewed English papers fulfilling the eligibility criteria in the final report. Besides, the following exclusion criteria were applied in the present study:

- Non-human studies, including animal experiments, in vitro observations, or papers with a limited report on COVID-19, and those without reference to this review's keywords.
- Papers with inaccessibility to their full texts.
- Any duplicated and suspicious outcomes in databases.

### Extraction of data

The authors’ names, publication date, article types (e.g., case reports), country of origin, sample size, gender, age, and clinical symptoms were recorded in an information sheet. This information was collected by two independent researchers and subsequently organized in tables. All authors cross-checked the selected articles to avoid any duplications or overlap in the content.

### Assessment of quality

This study adhered to the Preferred Reporting Items for Systematic Reviews and Meta-Analyses (PRISMA) checklist to ensure the quality and accuracy of selected papers and outcomes. Two independent researchers examined the quality of the articles and the probable risk of bias. A third researcher addressed any disagreement. The full text of selected articles was read, and the key findings are summarized in tables.

## Results

We identified a total of 56 relevant articles by title and abstract. Of 56 articles, 50 were related to the genomic variations in SARS-CoV-2. The included studies were conducted in 16 countries, and one of the articles was a report on multinational scientific collaborations [1] (Table 1 illustrates a summary of the findings). We summarized each study’s main findings in the two categories: genomic variants and other results.

**Table 1:** Comprehensive details of the included articles

Variants of COVID-19						
ID	The first author (reference)	Type of study	Country	Study population	Genomic variants	Other
1	Alouane [21]	Research article	Morocco	SARS-CoV-2 genomes	ORF1, Receptor binding domain (RBD), D614G, Q57H, T265I	A total of 3206 variant sites were detected compared to the reference genome Wuhan-Hu-1/2019. For each cluster, we identified different clades: cluster one containing two main clades A1a and B1 harboring mainly strains from Asia, North America, and Asia Europe, respectively. However, cluster 2 harbored three clades: B2, A2, A2a without a specific pattern
2	Al-Tawfiq Jaffar [22]	Letter to the editor	USA	COVID-19 patients	There were three genomic variants, termed A, B, and C, based on amino acid changes	–
3	Armengaud [23]	Opinion	France	COVID-19 patients	–	As expected for any RNA virus, over time, individuals are infected with SARS-CoV-2 variants that typically display some degree of genetic drift, compared with the first isolates of the virus obtained in Wuhan. The study of viral evolution indicates that genetic drift, mainly derived from genomic deletions, will almost inevitably attenuate the pathogenicity of viruses given enough time. The only hint at attenuation comes from an observation in Singapore, where some SARS-CoV-2 isolates turned out to have a 382-nucleotide deletion in ORF8 of the viral genome. This finding is of particular interest because of the omission of 29 nucleotides in ORF8 during the early spreading of SARS-CoV-1 in 2003. This 29-nucleotide deletion was demonstrated to attenuate viral replication when introduced in an infectious clone generated by reverse genetics
4	Bajaj [24]	Scientific correspondence	India	COVID-19 patients	–	The initial studies identified two major types of virulent SARS-CoV-2 circulating among the Chinese population. The aggressive form’s prevalence decreased after early January 2020, and the milder type has become standard due to selective human intervention. Two genomes available from India showed clustering closer to the strains prevalent in China to date. However, a unique mutation 24351C (A930V (T)) in the spike surface glycoprotein of Indian SARS-CoV-2 genome is absent in other strains from Wuhan, Italy, USA, and Nepal is reported
5	Bhowmik [20]	Research article	India	COVID-19 patients	Two groups (A and B) and further subgrouping (C, D and E) of the significant group A	SARS-CoV-2 genome is around 29,903 nucleotides and organized in the following order from 5' to 3': ORF1ab (replicas), structural spike glycoprotein (S), ORF3a protein, a structural envelope protein (E), structural membrane glycoprotein (M), ORF6 protein, ORF7a protein, ORF7b protein, ORF8 protein, structural nucleocapsid-phosphoprotein (N) and ORF10 protein. Studies have revealed that the highly mutable spike (S) protein of the virus is associated with the elevated human-to-human transmission rate through interaction with the host's ACE2 receptor
6	Biswas [25]	Letter to the editor	Bangladesh	COIVD-19 patients	This study analyzed 95 SARS-CoV-2 complete genome sequences obtained from Gen Bank, found 156 variants in total and 116 unique variants	Another analysis of 86 genomic sequences obtained from the GISAID database (https://www.gisaid.org/) identified three deletions in the genomes of SARS-CoV-2 from Japan, USA, and Australia, and 93 mutations over the entire SARS-CoV-2 genomes [3]. Several of these mutations were located in the receptor-binding domain (RBD) of the spike surface glycoprotein
7	Biswas [26]	Letter to the editor	Bangladesh	SARS-CoV-2 genomes	Receptor binding domain (RBD), RNA-dependent RNA polymerase (RdRp)	Several studies have so far been conducted utilizing sequencing data of SARS-CoV-2 obtained from publicly available repositories. One such study, analyzed 95 SARS-CoV-2 complete genome sequences obtained from Gen Bank, found 156 variants in total and 116 unique variants
8	Blackstone [27]	Commentary	USA	COIVD-19 patients	–	If a pathogen strain replicates too rapidly, the transmission might not occur before the host is debilitated. Fast-replicating pathogen strains may thus face extinction. Slow-replicating strains that cause mild or inapparent disease may allow a longer transmission window and persist in the host population
9	Cao [28]	Research article	China	COIVD-19 patients	Types A and C were only found outside East Asia, i.e., Europe and America, whereas type B was the most common type in East Asia	Results showed that mainland China strains' genomes were mostly distributed in Clade B and Clade undefined in the phylogenetic tree, with only 3.47% (5/144) found in Clade A. Also, Clades A2 (one case) and A2a (112 instances) contained no mainland China cases. In these clades, all cases came from 16 regions, mainly the Netherlands (65 cases), Switzerland (13 cases, and UK 13 cases), with only one case reported from Taiwan. Further analysis demonstrated that amino acid variation of the S protein at 614 (QHD43416.1: p.614D > G), i.e., the substitution of glutamic acid (D) with glycine (G) in the mutant protein, was found in strains within Clades A2 and A2a
10	Castillo [29]	Research article	Chile	Chilean COVID-19 patients	Three variants: S variant, G variant, V variant	According to prevalent SNPs, all genomes have been classified by amino acid changes in specific ORFs. The first three cases (20–1891820–1930320–19305) are classified as "S" type for the Chilean strains. Meanwhile, the fourth case (20–19731) is a "G" type, according to nucleotide substitutions in the positions 28 144 and 23 403
11	Everett [30]	Research article	USA	COIVD-19 patients	D614G, RNA-dependent RNA polymerase (RdRp) located on ORF1b	The D614G substitution has been proposed to promote infection of human cells, and this variant has spread globally at the expense of other genotypes
12	Forni [31]	Original article	Italy	SARS-CoV-2 genomes	D614G	Recent studies have indicated that the D614G variant, which is now prevalent worldwide, enhances viral infectivity
13	Forster [32]	Research article	Germany	COVID-19 patients	Three central variants: A variant B variant C variant	Node B is derived from A by two mutations: the synonymous mutation T8782C and the non-synonymous mutation C28144T changing a leucine to a serine. Type C differs from its parent type B by the non-synonymous mutation G26144T, which changes a glycine to a valine
14	Gómez-Carballa [33]	Research article	Spain	SARS-CoV-2 genomes	C8782T, C18060T, T28144C, C29095T	Sub-haplogroup A2 most likely originated in Europe from an Asian ancestor and gave rise to sub-clade A2a, which represents the major non-Asian outbreak, especially in Africa and Europe
15	Goren [34]	Letter to the editor	USA	COIVD-19 patients	TMPRSS2	Both SARS-CoV-2 and influenza are dependent on TMPRSS2 for infectivity, it is likely that SARS-CoV-2 will have a similar seasonal cycle; thus, the fall and winter are likely to see an increase in COVID-19 cases
16	Graudenzi [35]	Research article	Italy	COIVD-19 patients	–	Several mutations linked to low-rate mutational processes appear to transit to clonality in the population, eventually leading to the definition of new viral genotypes and to an increase of overall genomic diversity
17	Islam [36]	Original article	Bangladesh	COVID-19 patients	Three central variants A variant B variant C variant Clade S Clade V Clade G	–
18	Jain [37]	Research article	India	COIVD-19 patients	–	SARS-CoV-2 variants impact RT-PCR efficiency in detection. A total of 29 global SARS-CoV-2 genetic variants had a frequency ≥ 1%. The thermodynamic stability of the virus–primers complex gets perturbed. A number of recommended primer or probe sequences had high variant frequency
19	Jay [8]	Research article	France	COVID-19 patients	Genomic diversity of SARS-CoV-2 by next-generation sequencing (NGS)	For the first time, minority viral populations represented up to 1% during SARS-CoV-2 infection. Subspecies were different from one day to the next and between anatomical sites, suggesting that in vivo, this new coronavirus appears as a complex and dynamic distribution of variants
20	Joshi [38]	Research article	India	COVID-19 patients	C28854T and G25563T	From missense mutations found from Gujarat SARS-CoV-2 genomes, C28854T, deleterious mutation in the nucleocapsid (N) gene was significantly associated with mortality in patients. The other significant deleterious variant (G25563T) is found in patients located in Orf3a and has a potential role in viral pathogenesis. SARS-CoV-2 genomes from Gujarat are forming distinct clusters under the GH clade of GISAID
21	Junejo [39]	Review article	Pakistan	COVID-19 patients	ACE2, IL-10, TNF, VEGF	Elevated levels of cytokines and chemokines in COVID-19 patients including IL1 β, IL1RA, IL7, IL8, IL9, IL10, basic FGF2, GCSF, GMCSF, IFN γ, IP10, MCP1, MIP1 α, MIP1 β, PDGFB, TNF α, and VEGFA were observed. The increased pro-inflammatory cytokines, including IL2, IL7, IL10, GCSF, IP10, MCP1, MIP1 α, and TNF α, were responsible for disease severity. Many candidate genes, i.e., ACE2, IL-10, TNF, VEGF, are believed to be associated with ARDS development or outcome. In addition, the increased levels of IL-6 and IL-8 were confirmed to be associated with ARDS. Data collected on genetic evolution, receptor-binding, and pathogenesis have shown that bats most likely cause SARS-CoV by sequential recombination of bat SARS-CoVs
22	Korber [11]	Research article	USA	COVID-19 patients	D614G	An A-to-G nucleotide mutation causes the Spike D614G amino acid change at position 23,403 in the Wuhan reference strain. Three other mutations almost always accompany the D614G change: a C-to-T mutation in the 5′ UTR (position 241 relative to the Wuhan reference sequence), a silent C-to-T mutation at position 3,037, and a C-to-T mutation at position 14,408 that results in an amino acid change in RNA-dependent RNA polymerase (RdRp P323L). The haplotype comprising these four genetically linked mutations is now the globally dominant form
23	Kouriba [40]	Research article	Africa	COIVD-19 patients	M002593 and M002659	Analysis shows that both the early A (19B) and the later observed B (20A/C) clade are present in Mali, indicating multiple and independent introductions of SARS-CoV-2 to the Sahel region
24	Koyama [41]	Research article	USA	COVID-19 patients	D614G, L84S, L3606F, D448del and G392D	Several variants of the SARS-CoV-2 genome exist, and that the D614G clade has become the most common variant since December 2019. The authors identified six significant clades (that is, basal, D614G, L84S, L3606F, D448del, and G392D) and 14 subclades. Regarding the base changes, the C > T mutation was the most common distinct variants
25	Kozlovskaya [42]	Research article	Russia	COVID-19 patients	Amino acid substitutions	A specific set of seven nucleotide mutations using amino acid substitutions in spike protein S and nucleoprotein N, possibly affecting their properties
26	Laha [25]	Research article	India	COVID-19 patients	Nucleotide and amino acid sequences of ORF1ab, ORF3a, ORF6, ORF7a, ORF8, ORF10, envelop (E), membrane (M), nucleocapsid (N), and surface glycoprotein (S)	The surface glycoprotein, nucleocapsid, ORF1ab, and ORF8 showed frequent mutations, while envelope, membrane, ORF6, ORF7a, and ORF7b showed conservation in terms of amino acid substitutions. Some of the mutations across different proteins showed co-occurrences, suggesting their structural and/or functional interaction among different SARS-COV-2 proteins and their involvement in adaptability and viral transmission. Analysis of protein structure stability of surface glycoprotein mutants indicated the viability of specific variants and are more prone to be temporally and spatially distributed across the globe
27	Latini [43]	Research article	Italy	COVID-19 patients	ACE2, TMPRSS2, PCSK3, DPP4, and BSG genes	It is known that ACE2 acts as a receptor for this pathogen, but the viral entry into the target cell also depends on other proteins. In the PCSK3 gene, we observed a missense variant (c.893G > A) statistically more frequent compared to the EUR GnomAD reference population and a missense mutation (c.1906A > G) not found in the GnomAD database. In the TMPRSS2 gene, the authors observed a significant difference in c.331G > A, c.23G > T, and c.589G > A variant alleles in COVID-19 patients, compared to the corresponding allelic frequency in GnomAD. Genetic variants in these genes could influence the entry of the SARS-CoV-2. These data also support the hypothesis that host genetic variability may contribute to the variability in infection susceptibility and severity
28	Lau [44]	Research article	China	COVID-19 patients	Vero-E6 cells	The presence of a distinct motif in the S1/S2 junction region suggests the possible acquisition of the cleavage site(s) in the spike protein that promoted cross-species transmission. Through plaque purification of Vero-E6 cultured SARS-CoV-2, we found a series of variants that contain 15–30-bp deletions (Del–mut) or point mutations, respectively, at the S1/S2 junction. The unique cleavage motif promoting SARS-CoV-2 infection in humans may be under intense selective pressure, given that replication in permissive Vero-E6 cells leads to the loss of this adaptive function
29	Lee [45]	Research article	Japan	COIVD-19 patients	ACE2, TMPRSS2, TLR7	Genome‐wide association studies have identified genetic risk factors for severe COVID‐19 cases in a segment of chromosome 3 that involves six genes encoding three immune‐regulatory chemokine receptors and another three molecules. The risk haplotype seemed to be inherited from Neanderthals, suggesting genetic adaptation against pathogens in modern human evolution. Therefore, SARS‐CoV‐2 uses highly conserved molecules as its virion interaction, whereas its immune‐response appears to be genetically biased in individuals to some extent
30	Liu [46]	Research article	USA	COVID-19 patients	Single-nucleotide variants (SNVs)	Four signature groups of frequently occurred single-nucleotide variants (SNVs) were identified in over twenty-eight thousand high-quality and high-coverage SARS-CoV-2 complete genome sequences, representing different viral strains. Interestingly nucleotide substitutions among SARS-CoV-2 genomes tended to switch between bat RaTG13 coronavirus sequence and Wuhan-Hu-1 genome, indicating the higher genetic instability or tolerance of mutations on those sites or suggesting that major viral strains might exist between Wuhan-Hu-1 and RaTG13 coronavirus
31	Lokman [47]	Research article	Bangladesh	COVID-19 patients	N-terminal domain (NTD) and receptor-binding domain (RBD) and angiotensin-converting enzyme 2 (ACE2)	Spike glycoprotein is one of the major targets to be explored because of its role during coronaviruses' entry into host cells. Variations located at the N-terminal domain (NTD) and the receptor-binding domain (RBD) might alter the interaction of S protein with the host receptor angiotensin-converting enzyme 2 (ACE2)
32	Muhammad Ansari [48]	Research article	Indonesia	COVID-19 patients	SARS-CoV-2 spike glycoprotein gene sequences	Therefore, the coronavirus spike glycoprotein mediates membrane fusion and viral entry into host cells and is the primary target for many neutralizing antibodies. The spike glycoprotein has two domains, S1 and S2, where S1 is responsible for binding the virion to ACE2 on the host cell membrane 21. Several antiviral drugs and vaccines have been developed which target the spike glycoprotein. There was no significant difference between the SARS-CoV-2 spike glycoprotein gene sequences found in Indonesia and the Wuhan-Hu-1 isolate from China
33	Mukherjee [49]	Research article	India	COVID-19 patients	SARS-CoV-2 genome sequence	This study suggested a possible cross-talk between host RBPs-miRNAs and viral UTR variants in SARS-Cov-2 infection. The variations in the UTR regions and binding of host RBP to them remain mostly unaltered, which further influenced specific miRNAs’ functioning
34	Pachetti [50]	Review article	Italy	COVID-19 patients	SARS-CoV-2 genome sequence, RdRp gene	The virus is evolving, and European, North American, and Asian strains might coexist, each of them characterized by a different mutation pattern. The contribution of the mutated RdRp to this phenomenon needs to be investigated. To date, several drugs targeting RdRp enzymes are being employed for SARS-CoV-2 infection treatment. Some of them have a predicted binding moiety in a SARS-CoV-2 RdRp hydrophobic cleft adjacent to the 14,408 mutations we identified. Consequently, it is important to study and characterize SARS-CoV-2 RdRp mutation to assess possible drug-resistance viral phenotypes. It is also important to recognize whether the presence of some mutations might correlate with different SARS-CoV-2 mortality rates
35	Panchin [51]	Research article	Russia	COVID-19 patients	Single-nucleotide variations	Mutation patterns of SARS-CoV-2 have changed after transmission to humans. There are two remarkable observations regarding the excess of G–U transversions in SARS-CoV-2. One is the change in SARS-CoV-2 mutation rates after zoonotic transfer to humans since the proportion of G–U substitutions measured between the SARS-CoV-2 and the bat coronavirus RaTG13 is unremarkable. The second remarkable feature is that this excess of mutations is asymmetric: there is no similar effect for C–A mutations
36	Pardo-Seco [52]	Letter to the editor	Spain	COIVD-19 patients	C8782T–T28144C	Barcodes based on worldwide databases inevitably prioritize variants located at the basal nodes of the phylogeny, such that most representative genomes in these ancestral nodes are no longer in circulation. Consequently, coronavirus phylodynamics cannot be properly captured by universal genomic barcodes because most SARS-CoV-2 variation is generated in geographically restricted areas by the continuous introduction of domestic variants
37	Parlikar [53]	Research article	India	COIVD-19 patients	–	SARS-CoV-2 strains form a monophyletic clade distinct from SARS-CoV and Pangolin CoV that they are closest to the Bat CoV RaTG13 strain followed by Pangolin CoV, suggesting that SARS-CoV-2 evolved from a common ancestor putatively residing in bat or pangolin hosts. 44 protein-coding regions constitute the pan-genome for nineteen genus Betacoronavirus strains. Moreover, their pan-genome is open, highlighting the wide diversity provided by newly identified novel strains. Even members of subgenus Sarbecovirus are diverse relative to each other due to the relative presence of unique protein-coding regions orf3b, orf7b, orf8 and orf9b and orf10
38	Peñarrubia [54]	Research article	Spain	COVID-19 patients	The annotated single-nucleotide variations	Given that genetic variability in the SARS-CoV-2 genome is expected to increase based on the natural viral mutation and recombination rates, our results show that the combination of more than one assay target in real-time RT-PCR SARS-CoV-2 panels can mitigate the risk of loss of sensitivity or specificity
39	Peñarrubia [55]	Research article	Spain	SARS-CoV-2 genomes	Genomic variants detected in complementary binding regions of earliest available SARS-CoV-2 RT-PCR assays	Combination of more than one assay target in real-time RT-PCR SARS-CoV-2 panels can mitigate the risk of loss of sensitivity or specificity. In this regard, continuous monitoring of genomic variations is essential to provide a rapid response in case assay re-design is needed
40	Portelli [56]	Commentary	Australia	structural distribution of genetic variation in SARS-CoV-2 obtained from GISAID and COG-UK	SARS-CoV-2 spike protein's ACE2-receptor-binding domains (QHD43416 p). Asp614Gly), SARS-CoV-2 main proteinase domains (QHD43415_5)	That study developed a comprehensive online resource, COVID-3D, to enable the analysis and interpretation of variants detected in more than 125,000 SARS-CoV-2 genomic sequences. The SARS-CoV-2 spike protein binds h-ACE2, which mediates cell entry. Subsequently, the spike protein’s ACE2-receptor-binding domain has been the main target of most vaccine programs. Measures of selective pressure suggest that the spike protein is one of the viral proteins most tolerant of introducing mutations. Closer inspection indicates that substantial variation can be seen across the protein surface, including in predicted epitope regions in the receptor-binding domain. Of these variants, QHD43416 p.Asp614Gly is present in two-thirds of the sequenced strains, although its actual importance remains unclear, despite initial suggestions that it may increase transmissibility. It is identified several genes under strong purifying selection. These include the genes encoding helicase, RNA polymerase, NSP4, NSP9, and ExoN, which may serve as novel, promising drug targets with few circulating variants seen near the druggable pockets
41	Poterico [57]	Research article	Peru	691 SARS-CoV-2 complete viral genomes worldwide, including 30 genomes from South American countries in the GISAID database	ORF1a (G392D in nsp1, T708I in nsp2, A876T and A1043V in nsp3, N2894D and F3071Y in nsp4, G3334S in nsp5, L3606F in nsp6), ORF1b (P314L in nsp12), Spike protein (D614G, E1207V), ORF3a (Q57H, G196V, G251V), Membrane gene (T175M), ORF8 (L84S), N gene (D103Y, R191C, S197L, G238C, R203K, G204R)	Our results portray circulating SARS‐CoV‐2 South American strains coming from Europe, North America, and Oceania; and mostly belongs to Clade G. Infectivity and pathogenicity of SARS‐CoV‐2 is related to S protein, mainly due to the h‐ACE2 binding ridge structural changes of the RBD domain, on residues 482 to 485: Gly, Val, Glu, and Gly. Our report highlights two strains with novel variants on the S region, with no amino acid change in nt24022 (E1207E), whereas another non-synonymous alteration in nt25182 (E1207V), for Peru (EPI_ISL_415787) and Ecuador (EPI_ISL_417482), respectively. However, these changes seem far away from the critical region of S protein for h‐ACE2 affinity. Due to its prevalence, Clade G strains could be more contagious than other subtypes due to nucleotide changes in ORF1ab (nt8750) and N (nt29063) genes that enhance viral replication. We found 8 (8/30) variations in both of nt8782 and nt28144 positions. Conversely, other regions seem to be hotspots in South American strains, with 11(36.67%) of these portraying changes at 5′UTR (nt241), nsp3 (nt3037), nsp12 (nt14408), and N/ORF9 (nt28881, nt28882, and nt28883) regions. This is paramount because changes in nsp1, nsp3, and nsp5 could be related to some functions of the viral incubation period and immune response evasion of SARS‐CoV‐2. Amino acid alterations in both of these regions, such as G392D (nsp1), A876T, A1043 (nsp3), and nsp5 (G3334S); and should be tested in further studies. Strikingly, we identified four changes—nt15324 in ORF1ab (RdRp), nt26144 in E gene, nt28580, and nt28657 in the nucleocapsid gene—in the suggested regions for primer annealing for SARS‐CoV‐2 specific fragments identification, according to real-time RT‐PCR recommendations from the WHO. Moreover, viral genomes with alterations on 14 408 and 23 403 positions have been correlated with more mutations (3–4 per genome) than their counterparts without it. All South American viruses of Clade G analyzed in this report have mutations concomitantly on 14 408 and 23 403 nucleotidic positions
42	Romero [58]	Commentary	Peru	South American SARS‐CoV‐2 genome sequences in the SRA database	mutation N2894D in nsp4, non-synonymous mutation E1207E in the S gene	The de novo reassembly and mapped reads provided independent evidence to validate Poterico and Mestanza’s mutations based on the Peruvian SARS‐CoV‐2 genome. First, mutation N2894D in nsp4 (Table 1 in ref. 1) corresponding to a change from A to G in the nucleotide position 8945 occurs only in few reads (4 out of 33 mapped reads). It is not considered in the consensus sequence in the de novo reassembly. Thus, we should be cautious in considering this mutation as a real variant. Second, the authors reported a non-synonymous mutation E1207E in the S gene; this corresponds to T to C in the nucleotide position 24,022. Again, this mutation occurred only in 4 of 29 mapped reads, and it is not present in the consensus sequence. This evidence supports the necessity of using original sequence reads to verify if the previously described mutations in SARS‐CoV‐2 genomes are accurate, assembly artifacts, or sequencing errors
43	Sapoval [59]	Research article	USA	6928 SARS-CoV-2, 42 SARS-CoV-1, and 53 MERS genome datasets in addition to RNA-seq datasets from 151 COVID-19 positive patients	Intra-host single-nucleotide variants (iSNVs), consensus-level single-nucleotide polymorphisms (SNPs) and structural variants (SVs) in three clades V, S and G	Though RNA viruses mutate more rapidly than DNA viruses, there are a relatively small number of SNPs that differentiate the main SARS-CoV-2 clades that have spread throughout the world. First, the mutational profile of SARS-CoV-2 highlights iSNV and SNP similarity, albeit with high variability in C > T changes. Genes NSP6 and NSP10 are particularly enriched for T > C mutations, while NSP7 has an enrichment of A > C SNVs. Second, iSNV and SNP patterns in SARS-CoV-2 are more similar to MERS-CoV than SARS-CoV-1; except for significantly larger proportion of G > T changes in both iSNVs and SNPs. Third, a significant fraction of small indels fuel the genetic diversity of SARS-CoV-2. Fourth, the mutational spectra of the SNPs and iSNVs indicate that there is a complex interplay between endogenous SARS-CoV-2 mutational processes and host-dependent RNA editing. This observation is in line with several recent studies that propose APOBEC and ADAR deaminase activity as a likely driver of the C > T changes in the SARS-CoV-2 genomes. The study showed high sequence conservation within the NSP3 region, a region that is one of the most diverged from SARS-CoV-1 and MERS-CoV. The lower NSP3 mutations are due to its essential functional implications in viral replication, thus promising NSP3 as a good target for drug development. A number of convergent findings suggest de-mono-ADP-ribosylation of STAT1 by the SARS-CoV-2NSP3 as a putative cause of the cytokine storm observed in the most severe cases of COVID-19. Also, one deletion (at 28245 bp) was present in 10 samples (AF: 6%) in ORF8, a potentially important gene for viral adaptation to humans
44	Sarkar [60]	Research article	India	837 Indian SARS-CoV-2 strains	33 different mutations; 18 of which these were unique to India: S glycoprotein (L54F, K77M, R78M, D294D, E583D, Q677H), NSP3 (G716I, T749I, A994D, D1121G, S1197R), RdRP (A97V, L329I, G571S, V880I), NSP2 (S301F, G339S), and N (S194L)	Non-synonymous mutations were found to be 3.07 times more prevalent than synonymous mutations. The A2a clade was found to be dominant in India (71.34%), followed by A3 (23.29%) and B (5.36%), but a heterogeneous distribution was observed among various geographical regions. The A2a clade was highly predominant in East India, Western India, and Central India, whereas the A2a and A3 clades were nearly equal in prevalence in South and North India. D614G/S, a characteristic mutation of the A2 clade that was first reported in Germany, has been found to correlate strongly with high infectivity
45	Shen [61]	Research article	China	Bronchoalveolar lavage fluid samples from 8 patients with SARS-CoV-2, and 25 patients with community-acquired pneumonia (CAP), and 20 healthy controls for comparison	ORF1a, ORF1b, S, ORF3a, E, M, ORF6, ORF7a, ORF8, N, ORF10 mutations	The median number of intra-host variants was 1–4 in SARS-CoV-2-infected patients, ranged from 0 to 51 in different samples, suggesting a high evolution rate of the virus. The distribution of variants on genes was similar to those observed in the population data. However, very few intra-host variants were observed in the population as polymorphisms, implying either a bottleneck or purifying selection involved in the transmission of the virus or a consequence of the limited diversity represented in the current polymorphism data. Although recent evidence did not support the transmission of intra-host variants in a possible person-to-person spread, the risk should not be overlooked. Microbiota in SARS-CoV-2-infected patients were similar to those in CAP, either dominated by the pathogens or with elevated levels of oral and upper respiratory commensal bacteria. SARS-CoV-2 evolves in vivo after infection, affecting its virulence, infectivity, and transmissibility
46	Singh [62]	Research article	India	1,325 complete draft genomic sequences of SARS-CoV-2 and additional 279 CDS having partial genomes coding spike protein from NCBI database	RBD (A348V, V367F, A419S, T323I, A344S, R408I, G476S, V483A, H519Q, A520S, A522S, K529E, T323I, A344S, V367F, A419S, A522S, and K529E)	The significant variations in the predicted epitopes showing high antigenicity were A348V, V367F, and A419S in the receptor-binding domain (RBD). Other mutations observed within RBD exhibiting low antigenicity were T323I, A344S, R408I, G476S, V483A, H519Q, A520S, A522S, and K529E. The RBD T323I, A344S, V367F, A419S, A522S, and K529E are novel mutations reported for the first time in this study. Moreover, A930V and D936Y mutations were observed in the heptad repeat domain, and one mutation, D1168H, was noted in heptad repeat domain 2. S protein is the primary target for vaccine development, but several mutations were predicted in S protein's antigenic epitopes across all genomes available globally. The emergence of various mutations within a short period might result in conformational changes in the protein structure, suggesting that developing a universal vaccine may be a challenging task
47	Taboada [63]	Research article	Mexico	Covid-19 patients	present the full genome sequence for 17 SARS-CoV-2 isolates corresponding to the earliest sampled cases in Mexico	The authors reported that the initial virus strains introduced in Mexico came from Europe and the United States. The virus was circulating locally in the country as early as mid-March. They also found evidence for early local transmission of strains with an H49Y mutation in the Spike protein, which could be further used as a molecular marker to follow viral spread within the country and the region
48	Thielen [64]	Research article	USA	620 samples from the Johns Hopkins Health System collected between March 11–31, 2020; 143 of which was sequenced, generating 114 complete viral genomes	Identified a total of 153 unique, unambiguous single-nucleotide variants across all sequences (54 synonymous variants, 91 non- synonymous variants, 8 noncoding variants) compared to the Wuhan-Hu-1 SARS-CoV-2 reference genome	These genomes belong to all five major Next strain-defined clades, suggesting multiple introductions into the region and underscoring the diversity of the regional epidemic. We also found that clinically severe cases had genomes belonging to all of these clades. We found no clear correlation, but were limited by sample size. Similarly, patient phenotypes including sex, race, recent travel, symptoms, and comorbidities were represented across all five major phylogenetic clades, suggesting that susceptibility was independent of clade. The widely examined mutation in the viral spike protein (D614G) 28–30 is one of the key mutations differentiating the 19 and 20 clades. Notably, we see severe cases in both of these clades, though our dataset is underpowered to show significant correlations between viral genome mutations and disease severity. The diversity of virus genetics, clinical symptoms, and patient outcomes suggests that viral mutations are not the main driver of clinical presentation
49	Toyoshima [65]	Research article	Japan	Covid-19 patients	One thousand two hundred thirty-four mutations by comparing with the reference SARS-CoV-2 sequence	All replicating viruses, including coronavirus, continuously accumulate genomic mutations that persist due to natural selections. These mutations contribute to the enhancement of the ability of viral proliferation and infection and an escape from host immune attack
50	Ugurel [66]	Research article	Turkey	Covid-19 patients	Variations in SARS-CoV-2 genome	Despite some variations being in low-frequency rate in some continents, C14408T and A23403G variations on Nsp12 and S protein, respectively, were observed to be the most prominent variations all over the world, in general, and both cause missense mutations. It is also notable that most isolates carry C14408T and A23403 variations simultaneously, and also nearly all isolates carrying the G25563T variation on ORF3a, also carry C14408T and A23403 variations, although their location distributions are not similar
51	van Dorp [67]	Research article	UK	7710 SARS-CoV-2 assemblies flagged as “complete (> 29,000 bp)”, “high coverage only”, “low coverage excl” were downloaded from the GISAID Initiative EpiCoV platform as of April 19 2020	Identified 198 homoplasy positions in the SARS-CoV-2 genome alignment (0.67% of all sites) which was associated with 290 amino acid changes across all genomes; 232 non-synonymous and 58 synonymous mutations. Two non-synonymous mutations involved the introduction or removal of stop codons were found (*13402Y, *26152G)	One of the strongest homoplasies lies at site 11,083 in the SARS-CoV-2 genomes in a region of Orf1a encoding Nsp6. This site passed our stringent filtering criteria and was also present in our analysis of the SRA dataset. Interestingly, this region overlaps a putative immunogenic peptide predicted to result in both CD4+ and CD8+ T cell reactivity. More minor homoplasies among our top candidates, identified within Orf3a, also map to a predicted CD4 T cell epitope of note, we also identify a strong recurrent mutation in nucleotide position 21,575, corresponding to the SARS-CoV-2 spike protein (codon 5). While the spike protein is the known mediator of host cell entry, our detected homoplasy falls outside of the N-terminal and receptor-binding domains
52	Wang [68]	Research article	China	Covid-19 patients	Analyzed sequence variations along the SARS-CoV-2 genome	There may be selective mutations in SARS-COV-2, and it is necessary to avoid certain regions when designing primers and probes. The establishment of the reference sequence for SARS-CoV-2 could benefit not only the biological study of this virus but also diagnosis, clinical monitoring, and intervention of SARS-CoV-2 infection in the future
53	Xiao [69]	Research article	China	Clinical specimens (including throat swab, nasal swab, an anal swab, and sputum) obtained from confirmed COVID-19 cases at the First Affiliated Hospital of Guangzhou Medical University	SARS-CoV-2 genome sequences	This work offers practical guidance for genome sequencing and analyses of SARS-CoV-2 and other emerging viruses. We demonstrated that both amplicon and capture methods efficiently enriched SARS-CoV-2 content from clinical samples, while the enrichment efficiency of amplicon outran that of capture in more challenging samples
54	Yap [70]	Research article	Southeast Asia countries	142 complete sequences of SARS-CoV-2 from six of the SEA countries, including Cambodia (*n* = 1), Malaysia (*n* = 16), the Philippines/Philippines (*n* = 12), Singapore (*n* = 74), Thailand (*n* = 31) and Vietnam (*n* = 8)	ORF1a, ORF1b, S, M, E, ORF3a, ORF6, ORF7a, ORF8, ORF10	The authors focused on mutations that have emerged multiple times and identified 22 recurrent mutations in the SEA SARS-CoV-2 genomes. They also note that nearly 75% of the hits also overlap with candidate mutations, which may affect the phenotype of SARS-CoV-2 identified by Van Dorp *et al. *The current genomes studied showed phylogenetic relation with common recurrent mutations. Cluster I exhibited common recurrent mutation at 8782C > T in ORF1ab (*n* = 35). Forster et al*.* observed that the ancestral S variant with these two mutations at 8782C > T and 28144 T > C was predominantly identified in East Asia. Still, this variant outside of Asia was observed with striking, long mutational branch lengths. The G variant was rarely sampled in Asia but corresponded to the most frequent variant in Europe. In this study, G variants were identified in strains predominantly from Thailand, followed by Singapore and Vietnam. Cluster III belonged to clades outside the reported S, G, and V shared mutations distinguished clades, and at 6312C > A, 11083G > T, 13730C > T, and 19524C > T in ORF1ab, 23929C > T in spike, and 28311C > T in the N protein (*n* = 38). This variant was observed in strains from Malaysia, the Philippines, and Singapore from mid-March onwards. On April 19, 2020, a new cluster emerged from students returning to Malaysia from Indonesia
55	Zhang [18]	Research article	China	97 complete genomes of COVID-19 samples from GISAID	Three type-specific variants correspond to the genomic positions 8750, 28112, and 29063, respectively; the coordinates are referred to as the sequence MN938384.1	COVID-19 strains form two well-supported clades (genotype I, or Type I, and Type II). The two types' genomes mainly differ at three sites, which are 875028112 and 29063, based on MN938384.1's genome coordinates. Specifically, the nucleotides at the three areas are T, C, and T/C in Type I, and C, T, and C in Type II, respectively. Based on the nucleotide at the site 29063, the Type I strains can be further divided into Type IA and IB. The number of genomes belonging to type IA, IB, and is 10 18 and 69. Type II strains were likely evolved from Type I and are more prevalent than Type I among infected patients (68 Type II strains vs. 29 Type I strains in total). The outbreak of type II COVID-19 likely occurred in the Huanan market, while the initial transmission of the type I virus to humans probably happened at a different location in Wuhan. By analyzing the three genomic sites distinguishing Type I and Type II strains, they found that the synonymous changes at two of the three sites confer higher protein translational efficiencies in Type II strains than in Type I strains, which might explain why Type II strains are more prevalent, implying that Type II is more contagious (transmissible) than Type I
56	Zhu [71]	Research article	China	27,388 full-length COVID-19 genomes (collected from December, 2019 to May, 2020 from GISAID, NCBI, CoVdb and Viral Zone	9 new mutations; SNP-241 C > T, SNP-3037 C > T, SNP-8782 C > T, SNP-14408 C > T, SNP-23403 A > G, SNP-28144 T > C, SNP-28881 G > A, SNP-28882 G > A and SNP-28883 G > C; where the genome of the strain MN908947 is used as the reference	The 9 newly evolved SARS-CoV-2 single-nucleotide polymorphism (SNP) alleles reported, underwent a rapid increase(7 cases) 0 or decrease (2 cases) in their frequency for 30–80% in the initial four months, which are further confirmed by intra-host single-nucleotide variation (iSNV) analysis using raw sequence data including 8217 samples. The 9 SNPs are mostly (8/9) located in the coding region and are mainly (6/9) non-synonymous substitutions. They show a complete linkage in SNP pairs and belong to 3 different linkage groups, named LG_1–LG_3. Analyses in population genetics show signatures of adaptive selection toward the mutants in LG_1, but no signal of selection for LG_2. Population genetic analysis results on LG_3 show geological differentiation. Analyses on geographic COVID-19 cases and published clinical data provide evidence that the mutants in LG_1 and LG_3 benefit virus replication and those in LG_1 have a positive correlation with the disease severity in COVID-19-infected patients. The mutants in LG_2 show a bias toward mildness of the disease based on available public clinical data
	Novazzi [72]	Case report	Italy	66-year-old Italian male who tested positive for B.1.351. After returning from Malawi (Africa)	B.1.351 in PANGOLIN phylogeny or 20H/501Y.V2 in NextStrain phylogeny	Despite significant resistance to convalescent plasma and several mAbs, sera from human subjects vaccinated with mRNA-1273 led to 2.7 and a 6.4-fold geometric mean reduction in neutralization (but still 1:190) against K417N + E484K + N501Y + D614G or full B.1.351 Spike pseudovirus, respectively, when compared to the D614G VSV pseudovirus. Similarly, sera from human subjects vaccinated with BNT162b2 led to 0.81- to a 1.46-fold geometric mean reduction in neutralization against an E484K + N501Y + D614G spike pseudovirus finally, sera from persons vaccinated with one of 2 Chinese vaccines (BBIBP-CorV or recombinant dimeric RBD vaccine ZF2001) largely preserved neutralizing titres, with a slight reduction, against 501Y.V2 authentic virus
	Faria [73]	Reports	Brazil	Covid-19 patients	P.1	Lineage P.1, acquired 17 mutations, including a trio in the spike protein (K417T, E484K, and N501Y) associated with increased binding to the human ACE2 receptor
	Ferreira [74]	Research article	UK, India	Covid-19 patients	B.1.617	The defining mutations in B.1.617.1 spike are L452R and E484Q in the RBD that interacts with ACE2 and is the target of neutralizing antibodies

Studies have revealed that the highly mutable spike (S) protein of the virus is associated with the elevated human-to-human transmission rate through interaction with the host’s ACE2 receptor. A review of identified articles has shown three main genomic variants, including type A, type B, and type C. we also identified three clades including S, V, and G. Studies have demonstrated that the C14408T and A23403G alterations in the Nsp12 and S proteins are the most prominent alterations in the world, leading to miserable mutations.The spike D614G amino acid change has become the most common variant since December 2019. From missense mutations found from Gujarat SARS-CoV-2 genomes, C28854T, deleterious mutation in the nucleocapsid (N) gene was significantly associated with patients’ mortality. The other significant deleterious variant (G25563T) is found in patients located in Orf3a and has a potential role in viral pathogenesis.

## Discussion

Since the emergence of COVID-19, understanding the virus behavior has been received much attention from the scientific community. The different viral behavior has been attributed to the virus’s difference in types and strains [75]. Therefore, in the present review, we characterized the different variants of SARS-CoV-2 and discussed the findings in four sections, including the different types of SARS-CoV-2 variants, their effects on viral transmission, clinical manifestations, morbidity as well as mortality, and the other relevant findings.

### Different variants and strains

This review has focused on the different variations of SARS-CoV-2 and their impact on virus behavior. Alternation of the SARS-CoV-2 genome, through mutation and recombination, potentially leads to changes in the viral life cycle, including transitivity, cellular tropism, and severity of the disease. The diverse clinical outcomes in COVID-19 patients happen due to SARS-CoV-2 genome mutations. The mutation of single-stranded RNA viruses is much faster than the human genome's mutation rate, about 10^–6^–10^–4^ and 10^–8^, respectively [76, 77]. This leads to numerous quasi-species in each infected one, which may justify the observed difference in symptoms and disease severity [78]. Altered ACE2 binding interactions or shifted tissue tropism may happen due to a mutation among viral progeny that causes aggressive and immense infections [20].

Evolutionary benefits such as changing a primary epitope to escape from the host immune system or changing virulence factors to enhance transmission of the virus can occur due to gene mutations. Natural selection or vaccine selective pressure can cause these mutations and subsequently lead to new viral strains [79]. Preliminary studies at the beginning of the outbreak identified two major genotypes of SARS-CoV-2 among a Chinese population, type Ι, and type ΙΙ [18]. The prevalence of the aggressive form had decreased in the early months due to the start of treatment, and its mild form became the common variant [24].

Further studies reported the identification of three major variant types (A, B, C) of SARS-CoV-2, based on amino acid changes [22]. Forster et al. confirmed those three major variant types by phylogenetic analysis of 160 viral genomes [32]. Interestingly, variant A is the conventional type; type B viruses prevailed in East Asia, while both type A and C viruses have been dominant in America and Europe. After two mutations, including the synonymous mutation T8782C and the non-synonymous mutation C28144T, by replacing serine with leucine in type A, type B is formed. Type C is also derived from type B by the non-synonymous mutation G26144T, in which valine replaces glycine [32, 80, 81]. In other words, the S variant (Type A) with two mutations at 8782C>T and 28144 T>C was mainly identified in East Asia. Still, outside Asia, significant and long mutations were observed with the length of the branches. The G variant was dominant in Europe and was rare in Asia [70]. Bhowmik et al. reported two D and E subgrouping of the influential group A. Moreover, they stated that the SARS-CoV-2 genome is around 29,903 nucleotides. The highly mutable spike (S) protein of the virus is probably related to the increased human-to-human transmission rate through interaction with the host's ACE2 receptor [20]. Ugurel et al. reported C14408T variant on Nsp12 and A23403G variation on S protein, and both cause significant mutations and changes in virus variants worldwide [66].

Recent studies around the world have identified eight strains of SARS-CoV-2. However, they have a significant sequence similarity [50]. Also, Liu et al. have been recognized four distinct groups of common mononucleotide types (SNVs) in more than 28,000 high-quality, high-coverage SARS-CoV-2 complete genome sequences, demonstrating different viral strains [46]. These reports are consistent with the findings of two studies in Italy and the United States, where about 4–10 non-synonymous stable mutations were reported in the SARS genome [11, 50]. Eke, one of the mutations in S protein (D614G), has been seen repeatedly in Europe and the United States since the onset of the infection, apparently because it has dramatically increased the transmission ability of SARS-CoV-2. Thus, it became the most common variant [41, 56].

Although the mutation of the SARS-CoV-2 appears to be stable, consecutive consideration of virus mutations remains essential. A large study by Poterico and colleagues characterized two novel mutations in the S region across 691 complete viral genomes of SARS-CoV-2 from around the world. They also highlighted that the virus had acquired about 27 mutations, and most of South American countries’ trains are nearly related to European viral isolates [57]. Meanwhile, a unique mutation 24351C (A930V (T)) in the spike surface glycoprotein was reported in one of the Wuhan strains in India [24]. In a study conducted in Singapore, the cause of SARS attenuation was attributed to the 382-nucleotide deletion in ORF8 of the viral genome [23]. In a survey conducted in mid-March in Mexico, evidence of local translocation of strains with an H49Y mutation in Spike protein strains was reported [63]. According to the findings of Castello et al., the first three cases of ORF amino acid are classified as S type in position 28 144; nevertheless, the fourth case is a G type in position 23 403 [29].

### The effect of the variants on the viral transmission

Several studies have reported the association between the transmissibility and different variants and mutations [18, 20, 25, 27, 44, 51, 56, 57, 63, 75]. Some mutations facilitate the transmission of SARS-CoV-2 between animal species and humans. The G-U transversion excess might play a role in the bat to human transmission [51]. Besides, the S1/S2 junction region's specific motif may have caused the viral exchange between species [44].

Regarding the viral transmission between humans, some fundamental facts are noteworthy. The rapid viral replication might cause rapid morbidity and mortality and hinder the viral passage to healthy individuals. Viruses causing slower replications and asymptomatic or mild disease can allow the transmission for a more extended period [27]. Furthermore, mutations altering viral structure might increase virulence or helping the pathogen escape the immune system, resulting in higher transmission rates [75]. Type II SARS-CoV-2 strains possibly spreading through the Huanan seafood market were also considered more prevalent than type I viruses, probably due to being more contagious [18]. Several mutations are proposed to increase transmissibility [20, 57, 63, 75]. Variants possessing immensely mutable S proteins might be more contagious due to their interaction with the host ACE2 [20]. Concurrently, a specific mutation (D614G) in the S protein might speed up the viral transmission [75, 82]. The role of QHD43416 p.Asp614Gly variant in many strains is controversial and not fully understood [56, 82]. H49Y mutation in the S protein may also be responsible for local transmissions in earlier stages. It was proposed as a potential marker to trace the viral spreading between the countries and regions [63]. ORF1ab (nt8750) and N (nt29063) are also the identified responsible genes for the higher transmissibility of clade G strain [57].

### Variant effects on symptoms, morbidity, and mortality

Pneumonia and lung involvement is often the main clinical sign of COVID-19. Recent evidence also demonstrates gastrointestinal symptoms and asymptomatic infections [83]. The percentage of people infected with the coronavirus who remain asymptomatic during infection has not yet been accurately assessed and reported. Symptomatic patients often have clinical symptoms of fatigue, cough, nasal congestion, fever, and other signs of upper respiratory tract infections that usually appear after a week. The condition can develop into severe disease with dyspnea and severe chest symptoms [84, 85], and the respiratory tract infections are known as the primary clinical signs of COVID-19 [86].

In COVID-19, pneumonia usually manifests in the second or third week of symptomatic infection. Prominent signs of viral pneumonia include reduced oxygen saturation, blood gas deviations. Changes can be seen through chest X-rays and other imaging techniques, leading to the deterioration of vital signs and death. Lymphopenia (abnormally low level of lymphocytes in the blood) is common in these patients, and inflammatory markers (C-reactive protein and pro-inflammatory cytokines) could also increase [85, 87]. Furthermore, specific genetic mutations in the coronavirus can even increase mortality [85, 87].

### Other relevant findings

COVID-19 mortality rates differ substantially depending on the country. This difference in mortality rates depends on various factors in each country, including the adequacy of health care delivery, political decisions, and epidemiological characteristics of the affected population. The frequency of diagnostic and screening measures in asymptomatic or mildly symptomatic patients may also affect morbidity and mortality [88, 89].

Studies have demonstrated a steadfast and transparent pattern of age-based exponential enhancement in mortality, regardless of geographic area, in patients with COVID-19. Age-related mortality changes are relatively common for COVID-19 because other significant causes of mortality, especially chronic diseases such as cardiovascular disease, could also be increased by advanced ages [90]. Promislow et al. have shown that the mortality rate doubling time (MRDT) of all-cause fatality 9 years in the United States was close to that of COVID-19 in New York City [91]. In other words, there is no significant relationship between age and increased death in patients with COVID-19. However, many scientists and the media have paid particular attention to age as a risk factor for mortality in COVID-19. Nonetheless, the age-related pattern of death from COVID-19 is different from other respiratory viral infections. The pattern of morbidity and mortality is higher in the elderly than in young people [90].

## Limitations

Although this systematic review produces valuable knowledge regarding the COVID-19 variants and related morbidity and mortality, there were some shortcomings. First, the number of published reports is still limited. The knowledge regarding the different strains and variants and their effects on the symptoms, morbidity, and mortality is not entirely described yet. Furthermore, various countries ought to report their data to identify the worldwide distribution of these variants. Researchers might also strive to discover various novel mutations resulting in different viral behaviors in the future.

## Conclusion

Overall, researchers identified several SARS-CoV-2 variants changing clinical manifestations and increasing the transmissibility, morbidity, and mortality of COVID-19; however, many observations produced controversial results. Variants with asymptomatic disease or milder disease can increase their transmission by extending the duration of contact between sick and healthy people. Mutations causing increased virulence and immune escapes might also cause an elevated transmissibility level. As the vaccine inoculations are increasing worldwide, we encourage researching for potential mutations that might escape vaccine-induced immunity. The current practice and interventions should consider these findings to combat the COVID-19 pandemic and prevent related morbidity and mortality.

## Data Availability

The authors stated that all information provided in this article could be shared.
